# Phosphatidylinositol 4-phosphate 5-kinase Iγ_v6, a new splice variant found in rodents and humans

**DOI:** 10.1016/j.bbrc.2011.06.168

**Published:** 2011-07-29

**Authors:** Yang Xia, Robin F. Irvine, Maria-Luisa Giudici

**Affiliations:** Department of Pharmacology, Tennis Court Road, Cambridge CB2 1PD, UK

**Keywords:** PIP5KI, phosphatidylinositol 4-phosphate 5-kinase (type I phosphatidylinositol phosphate kinase), Phosphatidylinositol 4-phosphate 5-kinase, Phosphatidylinositol 4,5-bisphosphate, PIP2, Lipid kinase, Splice variant

## Abstract

Phosphatidylinositol 4-phosphate 5-kinase Iγ (PIP5KIγ) is subject to extensive C-terminal splice variation, with four variants, PIP5KIγ_v1, 2, 4 and 5, described in humans Schill and Anderson (2009) [7]. Here firstly, we report a new rodent splice variant, which includes the exon that was previously unique to the rodent neuron-specific PIP5KIγ93 Giudici et al. (2006) [6], but which omits the C-terminal exon of PIP5KIγ93; this new variant shows a wide tissue expression pattern in mouse. Secondly, we show that in humans there is an alternative splicing site 78 nucleotides from the start of exon 16c, such that humans additionally express both PIP5KIγ93 (which we now call PIP5KIγ_v3) specifically in brain and, again expressed more widely, the new variant described here, which we now name PIP5KIγ_v6.

## Highlights

► In this study we investigate splice variation in the lipid kinase PI4P 5-kinase Iγ. ► We report a new splice variant in rodents. ► We show that this new variant has a wide tissue distribution. ► We show, by identifying a new human splice site, that humans also express this variant. ► Finally, we show that another known splice variant, hitherto thought to be present only in rodents, is also found in humans.

## 1 Introduction

The synthesis of PI(4,5)P_2_ by Type I PtdIns4*P* 5-kinases (PIP5KIs) is the major route by which eukaryotes synthesise this multi-functional lipid. In addition to its obvious function as the precursor for both the PI-PLC and receptor-regulated PI 3-kinase pathways, PI(4,5)P_2_ has numerous other roles in its own right in, for example, secretion, endocytosis, F-actin and ion channel regulation [1,2]. Thus the regulation of the activity and localisation of the family of PIP5KIα, -Iβ and -Iγ, is inevitably going to be complex and sophisticated. The increasing complexity of regulatory mechanisms during evolution is aided by the appearance of splice variation, as it provides a greater variety of protein species independently of gene number [3]. PIP5KIs are no exception, with PIP5KIγ being the clearest example.

**Fig. 1:**
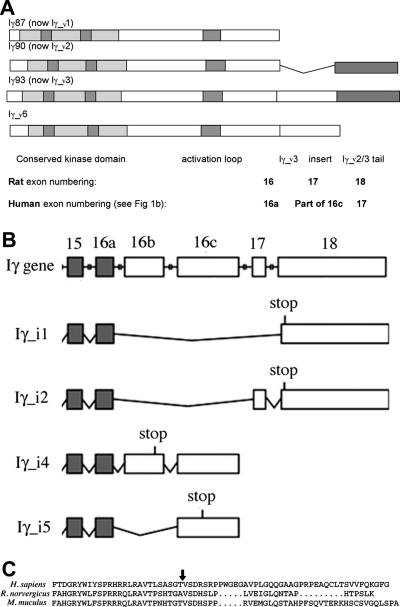
Splicing and exon patterns in human and rat. (A) Splicing patterns of PIP5KIγ in rat, including the new nomenclature from humans [7]. Equivalent human exon numbering is included for comparison. Note that the stop codon common to all these variants at the beginning of (rat) exon 19 is not shown. (B) Splicing patterns of PIP5KIγ in humans (adapted from Schill and Anderson [7]). (C) Amino acid sequences of human Iγ_i5-specific insert (B and Ref. [7]) with the homologous rat and mouse sequences below. The extra splice site that is used to generate human Iγ_i3 and Iγ_i6 (see text) is arrowed.

Ishihara et al. first cloned PIP5KIγ and showed that it has two splice variants with different C-termini, which they called A and B [4] (later known as Iγ87 and Iγ90, or Iγ635 and Iγ661). We previously described the discovery in a rat hippocampal cDNA library and the initial characterisation, including potential functions, of a new splice variant of PIP5KIγ [5], PIP5KIγC, since referred to as Iγ93 [6] (see Fig. 1A). A recent publication reported two further splice variants of PIP5KIγ specific to humans with unique C-termini largely unrelated to any of the above variants [7]. Both of these are also based on the Iγ87 core (see Fig. 1B – note that a different exon numbering system is used in humans). Apparently neither of these variants incorporates the exon homologous to rat 17, which is unique to Iγ93. Schill and Anderson [7] discussed the possibility that Iγ93 might be expressed in humans, and that because their new human variant Iγ_v5 is similar in the first part of its insert sequence to the Iγ93 insert, Iγ_i5 might replace the specific function of Iγ93 in non-neuronal tissues.

In this paper, firstly we report the discovery of a new splice variant in rodents, and secondly we demonstrate that both PIP5KIγ93 (now PIP5KIγ_v3, as suggested in Ref. [7] – see below) and this new variant are present in humans.

## 2 Materials and methods

### 2.1 RNA extraction and purification from the pituitary

Prior to the experiment, glass equipment was incubated in diethyl pyrocarbonate (DEPC, Sigma) overnight at 37 °C, followed by autoclaving. Other non-disposal equipment was cleaned using RNaseZAP (Sigma). Fresh or frozen (in RNAlater RNA stabilisation reagent, QIAGEN) pituitary tissues were homogenised in a Dounce homogeniser, using the RNeasy mini kit (QIAGEN). RNA yield was determined by spectrophotometry.

### 2.2 Reverse-transcription PCR

Access RT-PCR system (Promega) was used, with specific primers, to amplify the region of interest from RNA. Basic PCR and RT-PCR were carried out in one thermal reaction to produce double-stranded DNA fragments. RNA templates were obtained either from tissue extraction (see above) or commercially (mouse total RNA, Ambion). Primers were designed to span the junctions of two adjacent exons of the target, where possible, in order to avoid any potential contamination from genomic DNA. The pair of primers are designed to have similar GC content (>40%) and *T*_m_ (>78 °C).

### 2.3 Polymerase chain reaction

PCR primers were designed for rat, mouse or human as dictated by the cDNA library being probed, and are indicated schematically on each figure. The hippocampal and mouse tissue cDNA libraries were as described in Ref. [5]. The human templates were obtained commercially from Origene (First Strand cDNA of human tissues, Set 1).

Polymerase chain reaction (PCR) was performed using Taq DNA polymerase (Sigma) together with Pfu Turbo DNA polymerase (Stratagene) in a 4:1 M ratio. For a 50 μl reaction, 1 μl (5 U) of enzyme was supplemented with 50 ng DNA template, 10 μM of each primer, 200 μM dNTPs and 1× reaction buffer (50 mM KCl, 20 mM Tris–HCl pH 8.3). A typical thermal cycle consisted of 2 min at 95 °C, 25–30 cycles of 30 s at 95 °C, 1 min at 55 °C and 1 min at 72 °C. This was followed by 5 min at 72 °C, and 4 °C thereafter. Typical primer pairs were designed to be 18–22 bp in length, with a GC content of 50–60% and similar *T*_m_, between 55 and 60 °C.

**Fig. 2:**
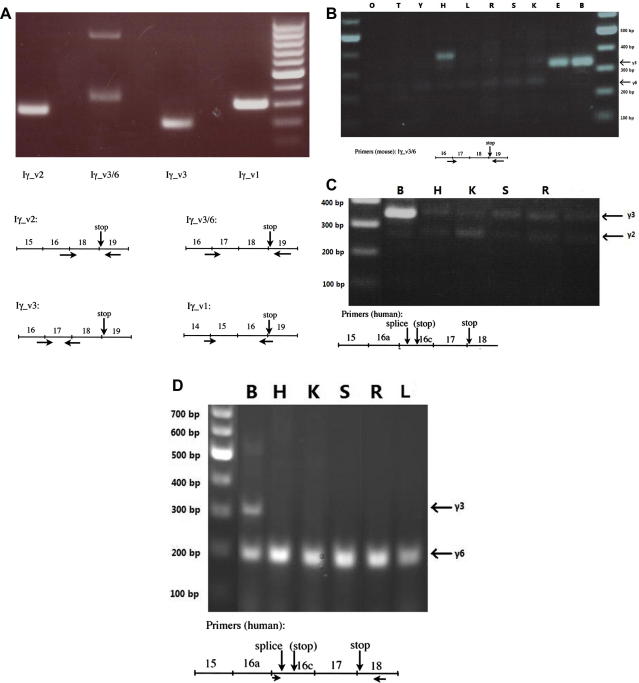
PCR amplification of human and rodent cDNA libraries. Primers used for A–D are depicted schematically (not to scale) below each figure. Note the different exon numbering in human versus rodent (see also Fig. 1A). For C and D (human) the alternative splice site within exon 16c that generates Iγ_v3 and Iγ_v6 (see Fig. 1C and text) is indicated by ‘splice’. The stop codon that ends Iγ_v5 (see Ref. [7]) is also indicated in brackets. (A) Rat hippocampal cDNA library [5] using rat-specific primers. (B) Mouse tissues total mRNA library [5] using mouse-specific primers O, ovary; T, testis; Y, thymus; H, heart; L, lung; R, liver; S, spleen; K, kidney; E, embryo; B, brain. The prominent band in heart was sequenced and is not a PIP5KI. (C) Human tissues cDNA library using human-specific primers. B, brain; H, heart; K, kidney; S, spleen; R, liver; L, leukocytes. Note that the lower band in brain and kidney is not Iγ_v6, but Iγ_v2 (see text). (D) Human tissues cDNA library using human-specific primers. For lettering see (C).

Actin controls were included in all experiments to ensure that the PCR took place. These are not shown in the figures because in all of these experiments PI5PKIγ-specific bands were identified and sequenced, and these bands in turn serve as positive controls, so that if another band is absent (e.g. Iγ_v3 in tissues other than brain or embryo – all panels of Fig. 2), this absence is not simply due to failed PCR.

### 2.4 Agarose gel electrophoresis

Agarose (0.7–1 g) was resuspended in 100 ml Tris-buffer saline (TBS, containing 50 mM Tris, 140 mM NaCl, pH 7.5), heated to dissolve and left to set in the gel tank at room temperature. DNA samples were loaded near the cathode in the loading dye (2.5% Ficoll-400, 11 mM EDTA, 3.3 mM Tris–HCl, 0.017% SDS, 0.015% bromophenol blue). A voltage of typically 80 V was applied till the blue frontline of the dye ran out of the gel. The separation pattern of the DNA bands could then be visualised and digitalised under the UV light.

### 2.5 Transfection

COS7 cells at 80–90% confluence were transiently transfected using the FuGene6 transfection reagent (Roche Molecular Biochemicals, USA), 24 h before imaging.

### 2.6 Live confocal imaging

Cells were imaged in fresh DMEM, either at room temperature or 37 °C. Image acquisition was achieved using a Leica CTR 6500 confocal microscope and Leica Application Suite software (2.4.1 build 6384). An Argon laser at 28 mW provided the source of illumination at 488 nm. A 40×, 1.25 NA oil-immersion objective was used, and GFP-fused kinases were visualised through a 495–535 nm filter. Images were collected in 1024 × 1024 pixels, as an average of 4 scans at 400 Hz.

## 3 Results and discussion

### 3.1 A new splice variant of PIP5KIγ in rodents

*In situ* hybridisation originally revealed that PIP5KIγ93 (now called Iγ_v3, see below) is expressed primarily in neurons [5]. Using the same RNA sequence specific to the PIP5KIγ93/Iγ_v3-specific exon 17 [5], we extended our *in situ* hybridisation studies to other parts of the mouse brain. We observed a strong signal from pituitary, and when we analyzed by RT-PCR a rat pituitary mRNA extract using primers specific for PIP5PIγ93/Iγ_v3, as well as the band of MW expected for that spliceoform we found an additional lower MW band. When sequenced this was found to be a new PIP5KIγ variant in which exon 17 (see Fig. 1A) is included, but exon 18 is omitted so that the stop codon at the beginning of exon 19 (also used for Iγ_v3 and not shown in Fig. 1A) follows immediately after exon 17.

### 3.2 Nomenclature of PIP5KIγ splice variants

As exons 17 and 18 are identical in size, this new pituitary splice variant has the same molecular weight as PIP5K Iγ90/PIP5KIγ661 (human PIP5KIγ_v2 in Ref. [7]), so naming it in the context of rodent nomenclature would be problematic. However, below we show that both the new variant and PIP5KIγ93 are expressed in humans. In their re-naming of human PIP5KIγ splice variants Schill and Anderson did not use ‘PIP5KIγ_v3’ in case PIP5KIγ93 was later found to be present in humans [7]. So we propose to follow their lead and use the name PIP5KIγ_v3 (abbreviated here to Iγ_v3) for PIP5KIγ93. Adopting this nomenclature then leads logically to calling our new variant PIP5KIγ_v6 (abbreviated to Iγ_v6). Note that we also follow Ref. [7] by using v for the nucleic acid (e.g. Iγ_v6) and i for the protein (e.g. Iγ_i6).

### 3.3 Pattern of PIP5KIγ_v6 expression

The PCR primers that we used to scan mouse tissue mRNA for the three variants Iγ_v1–3 [5] were from exon 16 (forward) to 19 (reverse), and we distinguished the three known spliceoforms on the basis of the molecular weight of the products [5]. However, as discussed above, Iγ_v2 would yield a product of exactly the same molecular weight as Iγ_v6 in such an experiment, so the whole issue of spliceoform distribution now needs re-examination with more specific primers and sequencing.

We therefore first re-analyzed the rat hippocampal cDNA library from which we originally cloned Iγ_v3 [5] with primers specific for each spliceoform (the primers used for Iγ_v6 will also amplify Iγ_v3). Fig. 2A shows that as we found before [5], Iγ_v1, 2 and 3 are all expressed in rat hippocampus. However, Iγ_v6 is not detectable in hippocampus; the very high MW band in lane 2 is not a PIP5KI, and the lower band was sequenced and confirmed to be from Iγ_v3.

We next did a full analysis of rat pituitary cDNA using the same primers and sequencing all the bands, and this revealed that pituitary expresses Iγ_v1, Iγ_v3 and Iγ_v6, but no detectable Iγ_v2 (not shown). We also analyzed the mouse pituitary cell line AtT-20 with the equivalent mouse-specific primers, and found a similar pattern of expression with again the notable absence of Iγ_v2. This latter variant is generally thought to be the most ubiquitously expressed of the PIP5KIγ spliceoforms, but its identification might sometimes have been complicated by the presence of Iγ_v6. To gain some insight into the expression pattern of Iγ_v6, we therefore re-analyzed the mouse tissue mRNA library that we previously used to explore tissue distribution of PI5PIγ variants [5], but using mouse Iγ_v3/6-specific primers. The data (Fig. 2B) confirm the high expression of Iγ_v3 in brain and its apparent absence in other tissues [5]. Lower MW bands, suggested to be Iγ_v6 on the basis of their MW, were detected in all tissues, and those bands from heart, liver, spleen and kidney were all sequenced and confirmed as being Iγ_v6. Overall these data suggest expression of Iγ_v6 in all the samples that are included in the library, implying that this spliceoform has a wide tissue distribution.

### 3.4 Human splicing

#### 3.4.1 Human PIP5KIγ_v3

Schill and Anderson [7] pointed out that the insert specific to human Iγ_i5 (exon 16c – see Fig. 1B) begins with a sequence of amino acids 75% identical to the Iγ_i3-specific 26 amino acids coded for by rat exon 17. In fact comparing the human and rat sequences reveals that the first 26 amino acids of the equivalent rat sequence is actually the Iγ_i3-specific insert (Ref. [5], see Fig. 1C). Thus Iγ_v3 could be conserved in humans if there was an alternative splicing after the 78 nucleotides coding for these 26 amino acids, such that the remainder of the exon 16c after these nucleotides up to the stop codon identified by Schill and Anderson [7] (see Fig. 1B and C) is either part of an exon (in Iγ_v5) or an intron (in Iγ_v3). This is very plausible because the human sequence here is ACTGTAAGT, where the ACT is the threonine codon that would end a human Iγ_v3 26 amino acid insert (arrow in Fig. 1C), and GTAAGT is a well documented 5′ alternative splice site consensus sequence [8,9]. We therefore tried to amplify Iγ_v3/6 from a human brain cDNA library from Origene (not shown) and also a human tissue cDNA library from Origene (see Section 2) that included brain (Fig. 2C). In both we found that the lower MW band in brain (and kidney, Fig. 2C) when sequenced was not Iγ_v6, but Iγ_v2, which we presume was amplified by some cross-hybridisation of the 16a/16c forward primer. However, the strong band consistent with Iγ_v3 by MW (Fig. 2C) was excised and confirmed by sequencing to be Iγ_v3, from which we conclude that humans do indeed express Iγ_v3.

#### 3.4.2 Human PIP5KIγ_v6

We then re-analyzed the human brain cDNA library with new primers (see Fig. 2D, the forward primer is within the 16c exon to avoid the spurious Iγ_v2 amplification) but were not able to detect Iγ_v6, only Iγ_v3 (not shown). This suggests that, consistent with the above data from mouse and rat, Iγ_v6 is not a major brain variant. Finally we used these primers on the human tissue cDNA library (Fig. 2D), and the lower MW bands from all the tissue lanes in Fig. 2D were sequenced and confirmed as being Iγ_v6 (note that in this library some Iγ_v6 is detected in brain, which may reflect different representation between libraries of regions such as pituitary, which is particularly Iγ_v6-rich). So, as in the mouse, in humans Iγ_v6 is apparently widespread in its distribution, while Iγ_v3 (again confirmed by sequencing of the higher MW band in Fig. 2D) is more brain-specific.

Thus overall we conclude that in humans there is an alternative splice site 78 residues into exon 16c that splices out the rest of the Iγ_v5 insert (see Fig. 1C), and then jumps either (a) to the start of exon 17 (Fig. 1B) to generate human Iγ_v3 (the stop codon is then at the start of human exon 18); or (b) straight to that stop codon in exon 18 to generate human Iγ_v6.

### 3.5 Properties of PIP5KIγ_i6

**Fig. 3:**
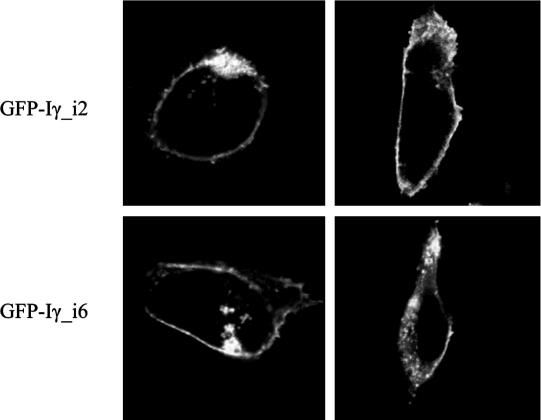
Localisation of expressed proteins. Representative images of COS cells transfected with GFP-tagged Iγ_i2 and Iγ_i6 (duplicates shown in each row).

The protein Iγ_i3 differs from Iγ_i1 and Iγ_i2 (and Iα and Iβ) in that when transfected into COS cells it is not plasma membrane-localised but largely intracellular [6]. Moreover, in HEK cells Iγ_i3 is on or near the plasma membrane, but unlike Iγ_i1 and Iγ_i2, it shows a large immobile fraction when analyzed by FRAP [6]. We do not yet understand the physiological significance of these empirical differences between transfected proteins, but to gain preliminary insight into Iγ_i6’s properties, we generated Iγ_i6 by introducing a stop codon into our GFP-tagged Iγ_v3 constructs immediately after the 26 amino acid insert, and then repeated these protocols. Fig. 3 shows that in COS cells the Iγ_i6 is apparently mostly plasma membrane-localised, and in these experiments was indistinguishable from Iγ_i2. We also found that in HEK cells Iγ_i6 has a FRAP profile indistinguishable from Iγ_i2 (not shown).

Finally, we generated an antibody to the 26 amino acid insert specific to Iγ_i3 and Iγ_i6, which specifically recognised these variants by both immunocytochemistry and Western blotting when they were transfected into cells. However, this antibody did not immunoprecipitate either splice variant, and moreover it did not show sufficient specificity or affinity to recognise unambiguously by Western blotting the endogenous proteins in tissue extracts of kidney or pituitary. The antibody also could not localise Iγ_i6 within kidney slices prepared as in Ref. [10], despite the apparent presence of the RNA in this tissue (Fig. 2B). A fuller understanding of the endogenous Iγ_i6 protein must therefore await better antibodies.

In conclusion, as we have discussed above, the existence of Iγ_v6 may be a complicating factor in studies which have investigated the distribution of Iγ_v2 if its presence is based on the MW of PCR products using primers such as those we used previously [5]. More importantly, our discovery of a sixth splice variant in humans of a PIP5KIγ that is similar to Iγ_v4 and Iγ_v5 in its broad expression pattern [7], but with an apparently different intracellular localisation from those two variants [7], highlights the diversity of potential PIP5KIγ forms, which is in turn probably a reflection of the remarkable diversity of PI(4,5)P2 functions.
